# Towards the microbial home: An overview of developments in next‐generation sustainable architecture

**DOI:** 10.1111/1751-7915.14256

**Published:** 2023-04-18

**Authors:** Rachel Armstrong

**Affiliations:** ^1^ Department of Architecture KU Leuven Ghent Campus Sint‐Lucas Belgium

## Abstract

Disruptive innovation is needed to raise the threshold of sustainable building performance, so that our buildings improve on net zero impacts and have a life‐promoting impact on the natural world. This article outlines a new approach to next‐generation sustainable architecture, which draws on the versatile metabolisms of microbes as a platform by incorporating microbial technologies and microbially produced materials into the practice of the built environment. The *regenerative architecture* arising from these interventions includes a broad range of advances from using new materials, to creating bioreceptive surfaces that promote life, and providing green, bio‐remediating energy from waste. Such innovations are presently reaching the marketplace as novel materials like Biocement® with lower embodied carbon than conventional materials that adopt microbially facilitated processes, and as novel utilities like PeePower® that transforms urine into electrical energy and bioreactor‐based building systems such as the pioneering BIQ building in Hamburg. While the field is still young, some of these products (e.g. mycelium biocomposites) are poised for uptake by the public–private economic axis to become mainstream within the building industry. Other developments are creating new economic opportunities for local maker communities that empower citizens and catalyse novel vernacular building practices. In particular, the activation of the *microbial commons* by the uptake of microbial technologies and materials through daily acts of living, ‘democratises’ resource harvesting (materials and energy) in ways that sustain life, and returns important decisions about how to run a home back to citizens. This disruptive move re‐centres the domestic‐commons economic axis to the heart of society, setting the stage for new vernacular architectures that support increasingly robust and resilient communities.

## Graphical abstract

The paper outlines a new approach to next‐generation sustainable architecture, which draws on the versatile metabolisms of microbes as a platform by incorporating microbial technologies and microbially produced materials into the practice of the built environment. The regenerative architecture arising from these interventions includes a broad range of advances from using new materials, to creating bioreceptive surfaces that promote life, and providing green, bio‐remediating energy from waste comprising a 'regenerative' architectural toolset.

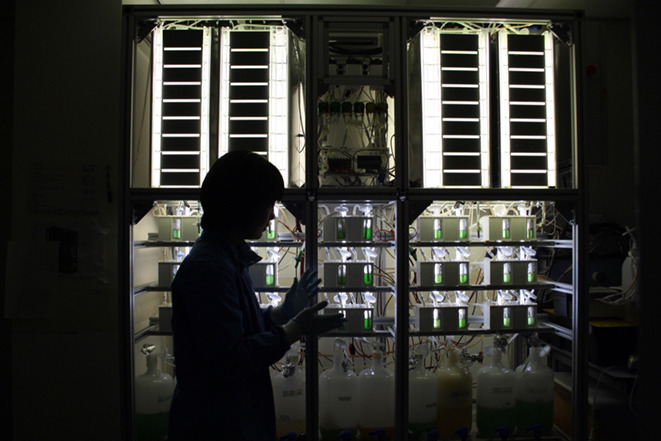

## ON ARCHITECTURAL IMPACT

The European Union's transition to a circular economy is a strategy to achieve its 2050 climate neutrality targets and halt biodiversity loss. Supporting initiatives along the entire product life cycle, the European Commission's 2020 circular economy action plan (CEAP) reduces pressure on natural resources while creating sustainable growth and jobs. However, to achieve complete circularity a new set of building materials, technologies and systems are needed.

This article asks whether changing our construction platform from the extractive, resource consuming ‘machines’ that characterize modern infrastructure, to ‘microbial technologies’—which refers to the use of microorganisms, such as bacteria and fungi, to produce goods or services—can radically alter the carbon footprint of the built environment, leading to strategies for a life‐promoting form of sustainable human development that meets the United Nation's Sustainable Development Goals (SDGs).

Small, versatile, metabolically robust, extremely diverse, superabundant, biologically alien (in comparison with multicellular organisms) microbes exist within an ethical grey zone (with respect to their relationship with humans^1^). Advances in biotechnology and insights from molecular biology provide architects and designers with a new perspective on the life‐promoting actions of microbes at the base of the biosphere, so they can be understood and incorporated into our homes and cities. Microbes comprise a ‘living’ technical platform for this new kind of design through their versatile metabolisms that catalyse the world's natural biogeochemical (Amils et al., 2023) and climate cycles (Cavicchioli et al., 2019). They can also provide various ‘goods’, through a range of biomolecules, that enrich ecosystems (Starke et al., 2019), which collectively, comprise a *microbial commons* that promotes biodiversity (Armstrong, 2023a; Dedeurwaerdere, 2010; Rest, 2021). As fundamentally environmental actors, microbes work within the carrying capacity of their different sites, metabolically transforming their surroundings into sites that are rich in bioavailable compounds. Drawing energy from their surroundings, collective microbial actions enliven environments, particularly through the soil. In this context, biotechnology has become a major source of inspiration and basis for establishing new type of relationship with nature through the emerging practice of BioDesign that draws on the properties of microbes in its processes to create fashion, textiles, furniture and architecture with radically new kinds of environmental relationships (Armstrong, 2022; Tsing et al., 2017; Myers & Antonelli, 2014). By working with living organisms, designers are engaging with the building blocks of the biosphere and are looking to cutting‐edge scientific developments to help them produce new materials and develop building services that convert organic waste into a range of valuable products in ways that significantly reduce our environmental and energetic footprints, enabling the practice of the built environment to improve on net zero and establish a regenerative approach to human development.

Today, the construction sector is responsible for over a third of the EU's total waste generation and 40% of our energy consumption is by buildings, which is more than industry or transport (World Green Building Council, 2019, 7; European Commission, 2020, 1). For buildings to be truly sustainable, all the different types of energy needed to make and run our homes must be considered. In the built environment, the most significant use of energy takes two main forms. The first kind of energy is used for building operations (which are all the active functions and services performed by buildings) is called operating energy, which is mostly used to run, maintain and manage our buildings. Most of this energy comes from fossil fuels to, for example, heat and cool our homes. This precious energy is often used inefficiently, as poorly insulated buildings squander heat, so more fossil fuel is needed to regulate indoor temperatures within acceptable limits of comfort. The second kind of energy is called embodied energy, which refers to the energy needed to extract resources, shape and compile them into a building. Manufacturing materials like bricks, glass, steel and concrete is very energy expensive, contributing 11% of total global carbon emissions. Modernity's favourite building material, concrete, is perhaps, the most energy‐expensive material. Together with cement, it is responsible for about 8% of global carbon emissions, which is more than double that from flying or shipping. Together they are responsible for the vast carbon footprints of the built environment, which could be mitigated by incorporating the co‐constitutive, regenerative processes of microbes into the production of materials and building services to establish domestic circular economies with multiple downstream consequences that can help us achieve a range of SDGs.

## THE MICROBIOLOGY AND ARCHITECTURAL CONTEXT

### Approach to building and construction

Growing our homes (or at least, substantial parts of them) is something that we have done since ancient times, which had a very low environmental impact. While modern practices position the idea of a growing, living building made of materials that are sown and cultivated, as improbable, prior to the industrial revolution all our homes and cities were developed in this way using locally available materials. In fact, most of the homes that were built in pre‐modern times have been reclaimed by nature and are revealed by aerial‐ and space‐archaeology using images taken from planes, drones and satellites to search for clues to the lost sites of past civilizations. The way we make our buildings today, however, means that growing our own homes seems unlikely—and perhaps a bit strange (Miller, 2017).

The first shelters were ‘found’, like natural caves, such as those that are still occupied by approximately 117 families in the town Kandovan, in Iran's East Azerbaijan Province. As people settled the land as farmers, specialist craftspeople could fashion local materials such as wood and stone harvested from quarries (which are open pits), to made buildings stronger and better buildings. The formation of more complex neighbourhoods, like villages, meant that people could claim their own land on which they built their homes, to produce dwellings like Kirkjubøargarður (King's Farm), the oldest inhabited wooden house which was built on the Faroe Islands during the eleventh century.

Owning your own land meant there was somewhere to grow and harvest materials for your home, whether you were making a new building or repairing an old one. Many kinds of homes were possible using a range of grown resources—from wooden beams to straw rooves. Each material was shaped by local craftspeople (artisans) in ways that made sense of their habitats using those available resources that could be sustainably sourced from the land, or in limited amounts from a quarry.

With the enclosure acts in England, between 1604 and 1914, the landed gentry took land from ordinary people, turning them into tenant farmers (rather than freeholders) who had to work on rented land. Enclosures caused poverty, homelessness and rural depopulation as workers lost their farms, their jobs and migrated to cities to find work. With no shared natural resources from where to grow materials to build their houses or produce their own food, workers were tenants once again, having to resort to the marketplace to rent their homes from landlords.

Rapid urbanization in the nineteenth century increased the demand for housing and construction needs were met by modern building techniques using resources obtained from elsewhere, which can be seen today in the two‐up, two‐down terraces, built from Accrington brick in Manchester's Trafford Park, the first industrial estate. Enabled by industrial processes, construction methods extracted resources from under the ground (mining), or the land (agriculture, forestry) beyond the city, rather than using local materials, creating scarcity, carbon emissions and waste. While local vernacular architectural practices optimized the relationship between natural energy sources, climate and local resources, enabling communities to be skilled in making their homes and buildings, these materials required large quantities of energy in their processing (kilning, smelting etc.) to turn them into building elements. Once made, these new building materials then needed to be transported to a distant building site.

As modern cities grew, the places and ways of making our modern buildings required far more materials and energy than in agrarian times, which were carried out by contractors aiming to making a profit, for landowners, who were also doing the same. To keep up with the demand, a new kind of fuel was used—fossil fuels (first, coal and then oil, which is even more energy dense than coal)—were burned to generate energy, which released a seemingly ‘clean’ gas (colourless, odourless) called carbon dioxide (chemical formula CO_2_). Barnabas Calder notes that *form follows fuel*, where the ready supply of effectively unlimited energy from fossil fuels could drive powerful machines (diggers, cranes) that enabled new methods of construction, expanding the repertoire of architecture (Calder, 2021). Associated new types of buildings were characterized by a particular *machine* aesthetic, as in the International Style, which were typified by rectilinear forms; light plane surfaces; open interior spaces; concrete, steel, sheet glass and cantilever constructions that sought to integrate traditional precedents with new social demands and technological possibilities. The convenience of instant, unlimited energy provided by fossil fuels, changed our expectations of buildings, with new kinds of structures (railway stations, skyscrapers and shopping malls) and services (motorway lighting, constant interior room temperature) in ways that were fundamentally resource‐hungry and energetically unsustainable. According to the Carbon Leadership Forum, our buildings are now responsible for about 40% of total global carbon emissions (Climate Leadership Forum, 2023), outstripping industry (20–30%) and transport (23%). Furthermore, greenhouse gas emissions resulting from—material extraction, manufacturing of construction products, construction and renovation of buildings—contribute 5–12% of total national greenhouse gas production (European Parliament, 2023, 1).

Over decades, the amount of carbon dioxide has built up in the Earth's atmosphere, which stops heat leaving from the planet's surface, causing an overall average rise in temperature, and leading to increasingly unpredictable weather patterns—a process called climate change, or more recently, the climate emergency. Owing to its substantial global carbon dioxide emissions, architecture is positioned at the heart of the challenge to reduce our fossil fuel dependency by upholding ambitious new approaches to achieve the transition to a circular economy (Global Alliance for Buildings and Construction, 2019).

### Impact of modern building techniques

The idea of ‘sustainable’ architecture was inspired during the 1970s energy crisis, when architects noticed that buildings which were enclosed in glass‐and‐steel boxes, required huge amounts of energy to power their heating and cooling systems.

Soon after these observations, sustainable development was defined as a concept in a landmark report called ‘Our Common Future’, also known as the Brundtland Report in 1987, which proposed:

Sustainable development is development that meets the needs of the present without compromising the ability of future generations to meet their own needs.

Sustainable buildings are those that are designed to help reduce the overall impact on the environment and human health during and after construction. Exactly how this is achieved depends on the architect, and the kinds of materials and systems available to them in preserving and protecting the natural resources that surround the project site. Cutting edge in sustainable concepts includes an ambition to reach net zero emissions, where buildings generate as much energy as they use. To ensure that all sectors of the global economy are working concordantly, the United Nations established *The 2030 Agenda for Sustainable Development*, which has been adopted by all UN Member States in 2015 working in a global partnership. The resultant 17 Sustainable Development Goals proposed are an urgent call for action by all countries, forming blueprint for peace and prosperity for people and the planet, now and into the future (United Nations, 2023).

In tackling these shared challenges, present trends in sustainable design for the practice of the built environment include ‘bio‐inspired’ approaches, which means the geometry (form) of the plant or animal is copied to make an ‘organic’—or nature‐based design statement. Bio‐inspired can also imply the building or object shares some qualities of the original organism (function). For example, the 300 St Mary Axe building in London, designed by Foster + Partners is better known as ‘The Gherkin’. Its mesh‐like exoskeleton mimics the structure of the Venus flower basket sea sponge, whereby the skyscraper's ventilation system is said to mimic how nutrients flow through the sea sponge, by directing airflow from the street level and open windows along the spiral frame to the offices. However, an ‘organic‐looking’ building is not necessarily a sustainable building as making these shapes requires huge amounts of concrete and steel. For example, Beijing's Olympic Stadium at the 2008 Olympic Games was designed by Herzog & De Meuron Architekten, Arup Sport and the China Architecture Design and Research Group, and configured in a form popularly known as the ‘Bird's Nest’. At the time, it was the largest steel structure, totalling 45,000 tons of the purest ever steel produced in China, used 10,000 tons of concrete and generated at least 130,000 tons of CO_2_ emissions in the process. According to the Climate Neutral Group, the capture of 1 tonne of CO_2_ emissions requires 50 trees growing for 1 year.

### Changing unsustainable architectural practices

In the current practice of the built environment, small changes can make relatively big impacts, and, in architectural design, microbes are being functionally integrated into the built environment for their biosynthetic abilities, through the incorporation of biofilms and certain types of microbial consortia into solid matrixes (e.g. agricultural wastes) or bioreactors, for cultivation. By altering environmental parameters, these microbes perform different kinds of work to collectively, turn waste streams into production systems for a range of materials and low power energy systems, establishing a platform for a circular resource economy (Timmis et al., 2019). Grown under the right conditions, these communities will grow at an exponential rate and can be significantly scaled up and combined with waste streams for making sustainable (circular) materials, or providing building services whose impacts align with, or even benefit, nature. Developments towards a microbial technological toolset and design portfolio to produce next‐generation sustainable buildings are discussed in the following sections. Examples do not propose to be exhaustive, as there is extensive scientific and design‐led research in this area, but rather discussion points are raised that survey state‐of‐the‐art developments.

### Microbial building materials

The following examples show how microbes can be cultivated and act as organic factories that produce new kinds of building materials that are sustainably grown from available resources often using waste streams, rather than being extracted from the ground. Two types of microbially produced materials are emerging: (i) those which attenuate the microbes (desiccation and heat treatments) and (ii) those which continue to cultivate microbes within the matrix of the material, which continues to exhibit multiple, ongoing functionalities.

#### Mycelium biocomposites

**FIGURE 1:**
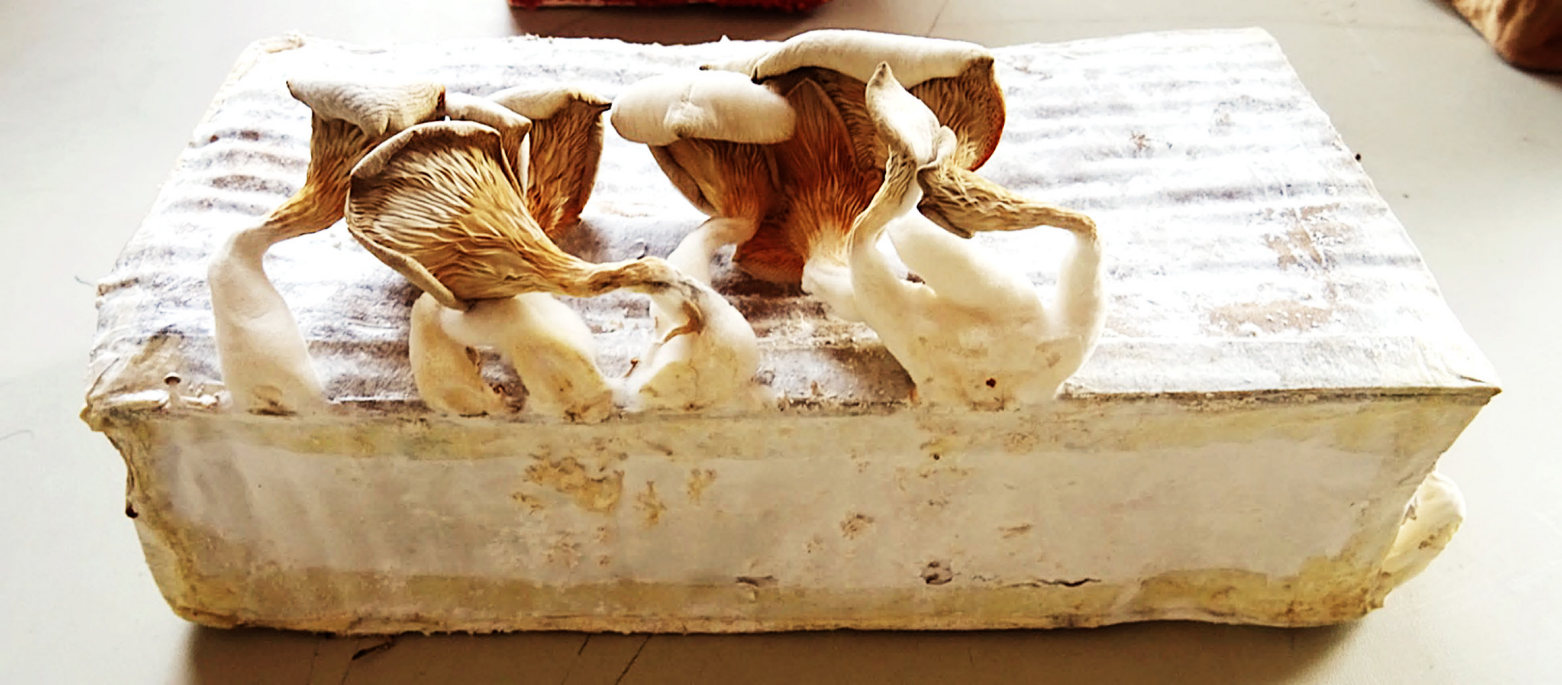
Mycelium biocomposite produced from cardboard and moulded in a Tupperware container (right) Samuel Hoornaerts, Wicked Home, Masters' Studio, Ghent, 2022.

The fine root‐like structures of fungi (mycelium) act like a *living glue* and can bind organic agricultural waste particles together (e.g. sugarcane bagasse, rice husks, cotton stalks and straw) to make a single, solid material called a *mycelium biocomposite* (Fairus et al., 2022; Sydor et al., 2022) (Figure 1). Part organic block, part fungal matrix, these versatile materials can be moulded, or 3D printed (Rahman et al., 2022), into a wide range of forms to become insulating bricks and even patchwork ‘tapestries’ of recycled materials—which keep our homes warm and insulate them against unwanted noise (Robertson et al., 2020; Yang et al., 2021).

The properties of material can be altered by selecting different types of fungi, changing the growing conditions, and using a final processing method to confer the material with a specific property (e.g. structural support, acoustic and thermal insulation) (Haneef et al., 2017; Li et al., 2022). Several companies such as MOGU (MOGU, 2023) and Grown.bio (Grown.Bio, 2023) are now selling these materials commercially for packaging, home insulation, soundproofing and as attractive interior panels for sustainable home design.

Mycelium biocomposites are relatively weak materials (0.1–0.2 MPa of compressive stress on average without mechanical compaction) and are thought to work best in compression for large‐scale structures, that is, as dome/vault, tower and column structural forms. They are also very lightweight, which provides an advantageous strength‐to‐weight ratios compared to concrete, which suggests that long‐span structures are possible. Several large‐scale pavilions have recently explored the potential of mycelium biocomposites for use in structural systems. Some designs engage mycelium biocomposites in a load‐bearing capacity, while other construction systems use the material as a surface or cladding application (Dessi‐Olive, 2022). The ‘Shell Mycelium’ in India (Areddia, 2017), the ‘Living Pavilion’ in the Netherlands (The Growing Pavilion, 2022) and the pavilion at the Rensselaer Polytechnique Institute, Troy, NY, USA (RPI, 2019), used mycelium biocomposites as cladding for wooden frame structures (Dessi‐Olive, 2022). By contrast, the 40 foot tower ‘Hy‐Fi’ installation by David Benjamin and The Living in 2014, engineered by ARUP (Saporta et al., 2015), sourced mycelium biocomposite ‘bricks’ from Ecovative that were supported by a wood and steel framework (Ecovative, 2023). Innovatively, the ‘MycoTree’ exhibited at the 2017 Seoul Biennale (Heisel et al., 2018) maximally exploited the structural capabilities of mycelium biocomposite units by placing them in compression‐only configurations. State‐of the art in construction employs the living properties of mycelium (Adamatzky et al., 2021) through techniques such as myco‐welding and fabric forming that can create complex yet efficiently formed wall structures from large building units grown in re‐usable formwork systems, which suggest that for the near future hybrid construction techniques and novel contributions from the mycelium itself will contribute to the broader canon of *myco‐fabrication* (Dessi‐Olive, 2022).

#### Biomineralization

Microorganisms naturally turn ions in solution into solid minerals via active cellular processes known as biomineralization (Simkiss & Wilbur, 2012) to provide structural support (Cosmidis & Benzerara, 2022), store energy (Jansson & Northen, 2010) and provide protection for microbes, as in thrombolites and stromatolites (Reid et al., 2000). Microbial‐induced calcite precipitation (MICP) is a widespread biochemical process in soils, caves, freshwater, marine sediments and hypersaline habitats that arises from metabolic interactions between diverse microbial communities with organic and/or inorganic compounds present in the environment including urea hydrolysis, denitrification, dissimilatory sulfate reduction and photosynthesis. Within the practice of the built environment, these biomineralization processes are induced by adding specific strains of bacteria, or fungi (Bindschedler et al., 2016) to calcium‐rich solutions, to induce calcite precipitation. Currently, MICP directed by urea hydrolysis, denitrification and dissimilatory sulfate reduction are the mainstay of bioconcrete production, with demonstrated improvements in mechanical strength and durability (Castro‐Alonso et al., 2019). Ureolytic biomineralization is used in a broad range of applications in construction and environmental protection, such as self‐healing concrete (Chen et al., 2020), bio‐bricks (Randall & Naidoo, 2018), dust stabilization (Aletayeb et al., 2021), ground improvement (Liu, Fan, et al., 2021; Liu, Zhang, et al., 2021) and bioremediation (Liu, Zhang, et al., 2021). A further benefit of using biomineralization in the production of building materials is that substrates such as granite, brick, marble and binders like fly ash, contain natural radioactivity, although this is typically very, very low but can result in unsafe levels of radon, which is a naturally occurring, hazardous radioactive gas that is formed from the decay of uranium in soil, rock and water (Eštoková & Palaščáková, 2013). The addition of bacteria and fungi that can express phosphatase activity in the presence of Uranium to biomineralize radionuclide, may provide a way of reducing the natural radioactivity found in building materials decays (Lopez‐Fernandez et al., 2020). Two products that are presently commercially available are outlined below.

- BioMason's first commercially available *Biocement®* product, Biolith, consists of approximately 85% natural aggregate and 15% biocement material. The product, which has the consistency of sandstone is grown from biomass, aggregate, nutrients and sand, by culturing them with *Sporosarcina pasteurii*, which absorb carbon dioxide from the air. The process can be described as simply as ‘brewing beer with sand’, which completely replaces the kiln and calcinator with a bioreactor, reducing the CO_2_ emissions compared to OPC by about 75% (Jølck & Perez, 2022). Since brick manufacturing makes 800,000 tonnes of CO_2_ emissions per year, biobricks are set to make a significant reduction in embodied carbon emissions (BioMason, 2023).
- *Bioconcrete* invented by Henk Jonkers at TU Delft, bioconcrete lasts longer than ordinary concrete, as it is seeded with hardy microbial spores of *Bacillus pseudofirmus and B. cohnii* (Sharma et al., 2017) that are activated by the entry of water when the concrete block develops a tiny fracture. On hydration, the spores spring to life and plug the fissure with mineral carbonate, extending the life of the material through this self‐healing characteristic (Jonkers & Schlangen, 2008; Stewart, 2016).

While the microbes used to produce commercial products are attenuated, in research laboratories, a generation of living building materials are being designed as functional, living *microbial* communities, which can grow and then be regenerated exponentially in response to physical switches. In a study conducted at the Living Materials Laboratory, University of Colorado Boulder, inert structural sand‐hydrogel scaffolds were used to provide support for *Synechococcus* sp. which, in turn, toughened the hydrogel matrix via calcium carbonate biomineralization, conferring the material with high fracture toughness. More broadly, *living building materials* represent a platform technology where biology can be leveraged to potentially deliver multiple functionalities to infrastructure materials by design (Heveran et al., 2020).

#### Bacterial cellulose

The ancient Chinese noted that when they made kombucha tea, a fermented beverage using a symbiotic colony of bacteria and yeast (SCOBY), the organisms became embedded within a thick fibrous mat composed of fine filaments, which could be harvested, dried and processed to be used in different ways (Gaggìa et al., 2018). This material is composed of polymers such as bacterial cellulose (BC), alginate and polyhydroxyalkanotes (PHAs) that exhibit enormous structural diversity and can be further modified using microbial biotechnology strategies combined with materials science to produce a range of materials with diverse properties, and non‐native features. Additionally, the next generation to these advanced materials will be conferred with smart functional properties, like sensing and responding to stimuli as well as being able to self‐repair, by incorporating living organisms into the fabrics using synthetic biology techniques (Hernández‐Arriaga et al., 2021). While the mainstay of applications are in the fashion industry, microbial polymers have many properties that are useful for next generation sustainable architecture being lightweight and being more tensile than plant cellulose. Likely architectural applications are facades, furniture and even for use in green walls, as a sustainable and environment‐friendly alternative to traditional materials like plastic and synthetic fibres (Fairs, 2014; Fernandes et al., 2019).

#### Microalgae‐based materials

Through their higher photosynthetic efficiency than in higher plants, owing to their wide range of pigments that can harvest more solar energy and a variety of carbon dioxide concentrating systems, microalgae can provide a sustainable source of bioproducts, including polysaccharides, lipids, pigments, proteins, vitamins, bioactive compounds and antioxidants (Plaza et al., 2009). At the micro‐level, free‐living microalgae make excellent substrates for generating bioinks, which are embedded in a gel, so they can used in 3D printing to oxygenate the medium and create the final shapes of different applications, like artificial leaves, photosynthetic skins or photosynthetic bio‐garments (Maharjan et al., 2020). In architecture, BioSolar Leaf panels that harness microalgae to turn solar energy into food are being trialled on a rooftop system in London with the aim to combat air pollution. The *synthetic leaves* are arranged in large solar panel‐like structures, where each leaf contains microalgae, phytoplankton and diatoms that are suspended in a durable and long‐lasting silk material. One acre of the BioSolar Leaf cultivation system is thought to remove carbon dioxide and produce breathable oxygen at a rate equivalent to a hundred trees from the surface of just one tree and can be installed on land, buildings or other infrastructure to improve the quality of the atmosphere. Trial data that prove the efficacy of the proposal are not yet available (Evanson, 2019). A range of making practices are emerging that integrate living photosynthetic microalgae with experimental methods of digital fabrication for sequestering CO2 from internal building settings (Crawford et al., 2022). These new biocomposites incorporate flexible textile substrates, that is, cotton, hessian, polyester and canvas, to provide a range of algae laden matrices that continue to develop and change during the useful part of the material's life cycle (Stefanova et al., 2021).

#### Water‐absorbing microbial spores [actuators]

*Bacillus subtilis* microbial spores are hygroscopic and can be turned into a solution for printing on fabrics to form a heterogeneous multilayered structure that responds to moisture by changing its shape. With the right kind of microbes, geometry and material, the water absorption, for example, from human sweat can be used to make biohybrid films that can reversibly change shape within a few seconds owing to the entry of water. Presently, these spore‐based shape‐change materials are not only being developed for sports clothes but are also being considered as architectural textiles for outdoor structures that are actuated by water (Media Lab, 2015; Wang et al., 2017).

#### Microbial pigments

Microorganisms produce a range of major pigments that are widely used in pharmaceuticals, food, paints and textile industries (Agarwal et al., 2023). Melanin is a dark, protective pigment found in all kingdoms of life, which absorbs harmful ultraviolet (UV) rays from the sun (which accounts for its darkness) and scavenges toxic *free radicals*, which are unstable atoms that can cause tissue damage when their levels become too high, and so, protects cells from damage. Fungi use melanin to harvest energy for cell growth and survive high temperatures, chemical stresses and biochemical threats (Casadevall et al., 2017; Cordero et al., 2022; Cordero & Casadevall, 2019; Mattoon et al., 2021). The integration of melanin‐producing fungi into biocomposites, or bio‐degradable substrates like bioplastics is of great interest to the production of next‐generation sustainable building elements, especially when the organism is still living for the production of claddings that harvest energy or protect inhabitants from radiation, as a means of mediating between the internal and external environments of a building—just like a ‘living’ skin does (Aouf, 2019).

#### Bioreceptivity

Materials that can host and support the natural growth and colonization of surfaces by environmentally beneficial microbes and small plant species, without the need for additional layers of support are bioreceptive. Properties are conferred by their material composition and physical characteristics, especially with respect to their ability to promote microbial attachment. Bioreceptive characteristics include rough surfaces, which form microclimates and provide attachments, porosity and patterns that assist the flow of water encouraging microbial growth and surface succession by simple plants (Mustafa et al., 2021). Without design intervention, plants like microalgae, mosses, liverworts and lichens will spontaneously colonize facades but are typically considered a nuisance, although they have significant environmental impacts (Elbert et al., 2012). For example, moss can absorb 1 kg of carbon dioxide per each half square metre (Freeman et al., 2012), resulting in 14 billion tonnes of carbon dioxide being removed from the atmosphere and 50 million tonnes of nitrogen fixed worldwide per year (Elbert et al., 2012). In 2018, the installation *Subculture: Microbial Metrics and the Multi‐Species City*, by David Benjamin (The Living), Kevin Slavin and Elizabeth Hénaff at the Storefront for Art and Architecture Gallery, cladded the building with wooden panels that were cut with a concentric circular pattern (macro shapes) to collect the microbial life from the immediate environment for its downstream analysis using small‐scale genetic sequencing devices. In this way, the façade became a living environment and analytic laboratory, turning the whole building in a ‘urban metagenomic sensor’. Wood was selected as the ideal bioreceptive material owing to its molecular composition, being comprised of micro shapes (at the level of the wood grain) and macro shapes (at the level of geometric design) (Storefront for Art and Architecture, 2019). While bioreceptive materials are not specifically incorporating microbes into their substance, they recognize their beneficial contributions to ecosystems services, which—with the loss of natural soils—are increasingly vital for healthy urban spaces.

#### Combined microbial biocomposite materials

Natural materials are never homogeneous, so different microbial processes can be combined to create hybrid materials, whose performance is more than the sum of their ingredients. For example, BioMason (which makes Biocement®) is working with Evocative (which produces mycelium biocomposites) to make a stunning new kind of stone that is grown from agricultural waste, mushrooms, bacteria and sand (Grown.Bio, 2016). Many new types of biocomposites will be generated in this way—some attenuated, some ‘living’—to provide an increasingly diverse material portfolio that can be customized to local needs, employ prevalent microbial strains and respond to specific environments, which will accelerate design‐led innovation in next‐generation sustainable buildings.

### Microbial building services

Although new materials that are non‐extractive and incorporate waste streams in their production can reduce embodied energy, the bigger challenge is in reducing emissions from building operations. More distributed forms of energy and resource harvesting are being developed within the context of the built environment by harnessing the metabolic processing power of microbes. Although raw energy outputs are not yet entirely competitive with wind and conventional solar panels, microbial technologies are unmatched with their bioremediating capacities and possess an unmatched regenerative capacity that is capable of sainting waste stream, generating new resources and promoting biodiversity. Currently, a range of bioreactor systems are poised for wider integration into the urban fabric and are discussed in the following sections:

#### Microalgae building facades

**FIGURE 2:**
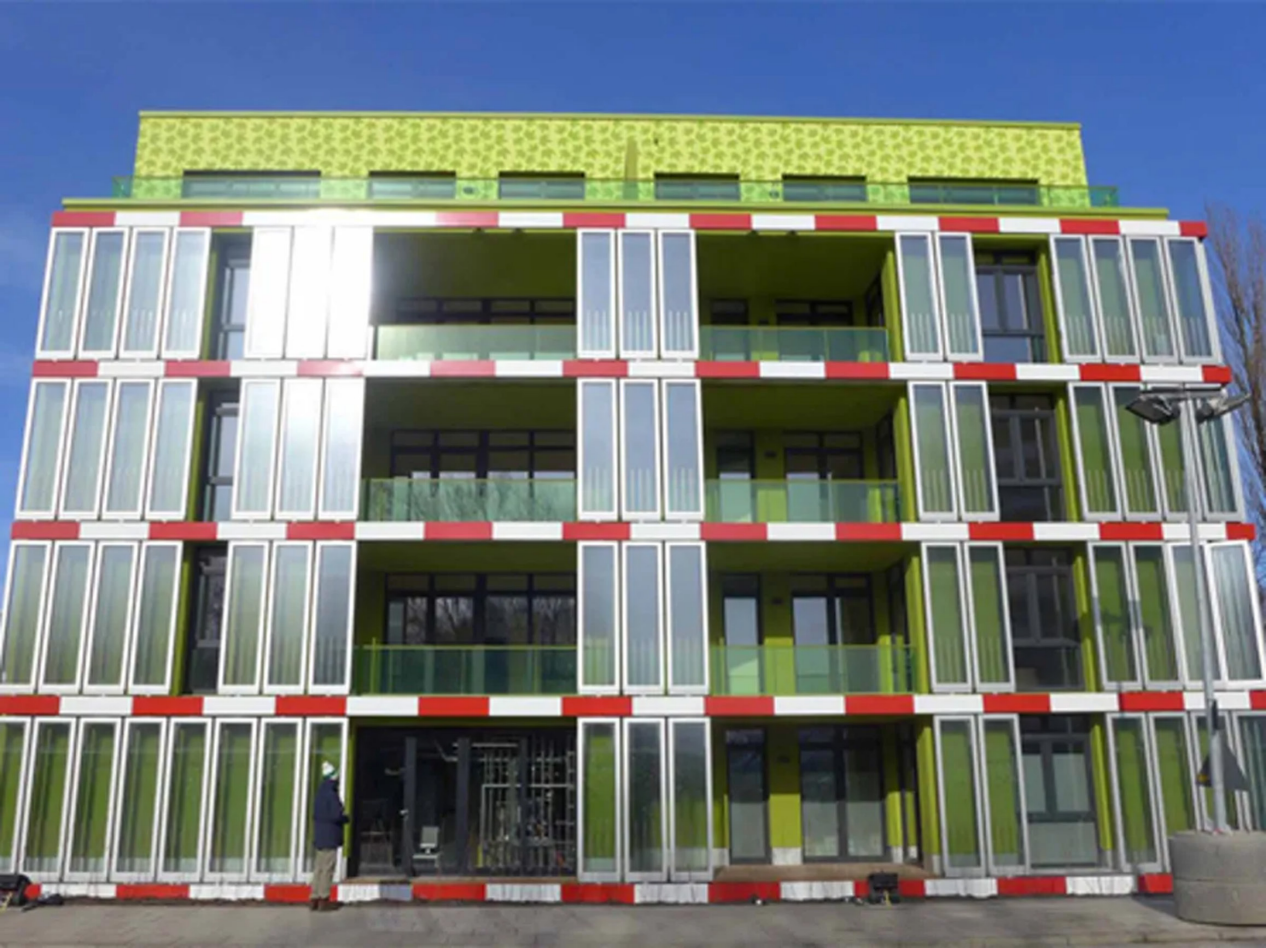
*BIQ House*, the world's first building powered by algae, Hamburg, Germany, courtesy of Colt International, Arup, SSC GmbH, *2011*.

Microalgae cultivation can be scaled up to the macro level in photobioreactors (PBRs), where their products can be harvested and used as sustainable resources, for example, biofuels and biomass, resulting in energy saving, reduced carbon dioxide emissions, oxygen generation, biofuel production, wastewater treatment and solar heat absorption. Such bioreactors can be incorporated into urban space—not just to produce novel, functional building materials but also to power building operations by producing biomass for biodigesters or extracting biofuel from the microalgae—which is more costly. Buildings with integrated PBRs propose to reduce energy consumption by a combination of thermal insulation, shading, solar collection and light‐to‐biomass conversion, with savings up to 30% due to reduced heating, cooling, ventilation and lighting loads (Sedighi et al., 2023). The BIQ (Bio Intelligent Quotient) building in Hamburg is a is a 15 storey concrete apartment building (Build Up, 2012) whose facade is wrapped in flat glass PBRs containing microalgae spanning 200 m^2^ on two sides (Figure 2). The algae façade sequesters approximately 16 kg of CO_2_ per day with a biomass production equivalent to 30 kWh/m^2^ year and heat production of 150 kWh/m^2^ year, with a net annual energy supply of about 4500 kW/h of electricity more than an average household consumes in a year (3500 kW/h per year) (Arup, 2022; BIQ, 2011; CE&CR, 2023).

While integrated PBRs offer some advantages in reducing energy for building services, the technology is still in pilot forms and some major challenges need to be addressed before the technology offers net‐zero services, which include—long‐term performance analyses in energy efficiency and effective CO_2_ sequestration, characterization of thermal and acoustic insulation, controlling indoor colour due to variation of algae culture density and algae medium discolouring, environmental durability of the panels, protocols for maintenance services, expense of construction, high‐power consumption needed for pumps and artificial light sources (in combination with partial exposure to sunlight) and negative environmental effects such as possible toxins and malodour (Elrayies, 2018; Kunjapur & Eldridge, 2010; Talaei et al., 2022; Wilkinson et al., 2016). In general, the high cost of installation and maintenance is offset by the possibility of long‐term environmental benefits, but these are not systematically proven.

#### Microbial fuel cells

Michael Cresse Potter designed a kind of ‘living battery’ in 1911, which produced several hundred millivolts of energy from the *Saccharomyces* yeast. This microbial fuel cell (MFC) captured electrons from the metabolizing yeast as it converted the chemical energy of sugar, using electrodes which flowed into an external circuit to provide electrical power for as long as the microbes continued to be fed. Although Potter also noted that *Escherichia coli* bacteria produced electricity under these circumstances but, microbial culturing was in its earliest days as a science, and he had very little success with these organisms or any other bacterial species (Potter, 1911). Previously, MFCs used pure culture organisms that required the addition of a synthetic mediator to facilitate electron transfer to the anode (Gasteiger et al., 2003), but number of microorganisms are electrogenic and can produce their own electron shuttles (Holmes & Nevin, 2004) and include *Shewanella* (Gorby et al., 2006; Wang et al., 2013) *Geobacter* (Bond & Lovley, 2003; Nevin et al., 2008) species or, mixed culture, natural waste water sources, brewery waste water and sediments from the sea and lakes (Feng et al., 2008; Rabaey & Verstraete, 2005). The organization of MFCs is like a chemical battery with an anode and cathode, but instead of using inert solutions, the MFC cultivates an anaerobic biofilm in the anode, which is separated from the cathode by a proton‐exchange membrane. To produce energy, the biofilm metabolizes an organic ‘feedstock’, which can be urine, grey water, black water or any kind of liquid organic waste. While bacteria in the anode digest the organic feedstock, electrons are released from the substrate oxidation in the anode compartment (the negative terminal) and are transferred to the cathode compartment (the positive terminal) through a conductive material, which are captured by electrodes, to generate an electrical current sufficient for powering electronic devices. Simultaneously, protons pass through the membrane where they combine with oxygen to produce water. While they are making bioelectricity, the anaerobic biofilms in the MFCs are also recovering nutrients, making biofertilizer (in the stabilized sludge they produce, which is a rich source of nitrogen and phosphate), treating wastewater and killing pathogens—just like natural soil biofilms—and unlike modern utilities, which are designed to process one type of resource at a time, MFCs are parallel processors.

Breakthroughs in MFC technology are multiple as, there are many elements that can be altered to change the overall performance of the cell from—microbial selection, to genetic and molecular engineering, electrode materials and membrane design. In the last couple of decades, field trials for MFCs in the treatment of wastewater streams have been conducted including a range of PeePower® urinals developed by Ioannis Ieropoulos at Southampton University, funded by The Bill & Melinda Gates Foundation, and Oxfam, as part of the reinvention of the toilet challenge for use in refugee camps and schools (Walter et al., 2018). These toilets provide basic lighting that keeps people safe at night, as well as providing water and sanitation. PeePower® urinals have also been piloted in a Western context in the last 5 years at the pop‐up Glastonbury festival where revellers can exchange their urine for—charging their mobile phones, playing computer games and powering some of the festival screens.

**FIGURE 3:**
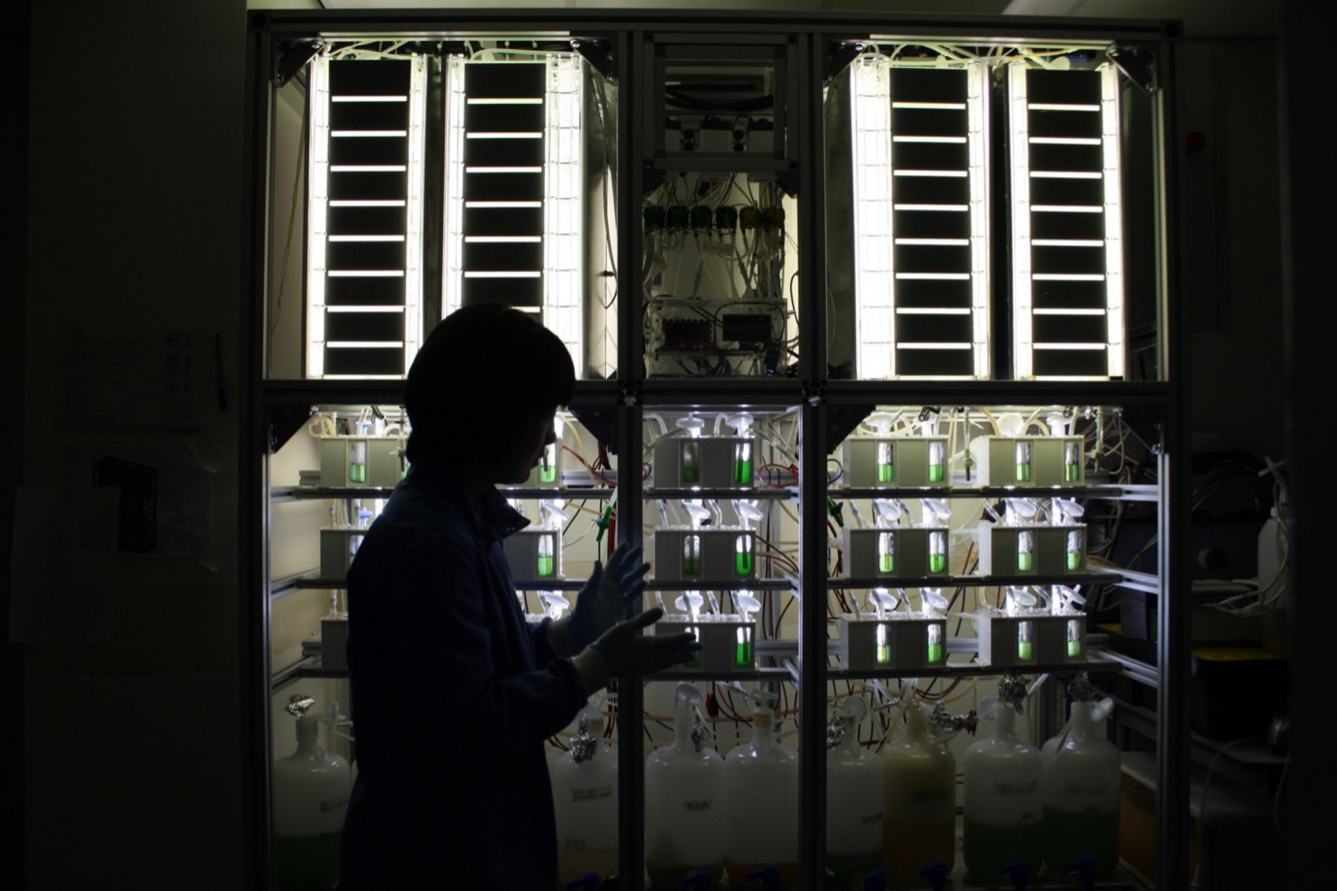
The Living Architecture bioreactor ‘wall’ courtesy of the Living Architecture project, 2019.

MFCs have also been sequenced with different bioreactors, whereby the microbial products could be treated, produced and reclaimed as a circular system within a household setting (Armstrong et al., 2017). The EU‐funded Living Architecture project successfully proved the principle that different bioreactors (photobioreactors, microbial fuel cells and synthetic bioreactors with engineered microbes) could exchange different types of nutrients within household liquid wastes and turn them into electrical energy 4–5 mW/unit and biomass (Figure 3). While the power output for this system is low, it invites the development of low‐powered direct current (DC) electronic systems instead of modernity's high power (230 V) alternating current (AC) supplies.

**FIGURE 4:**
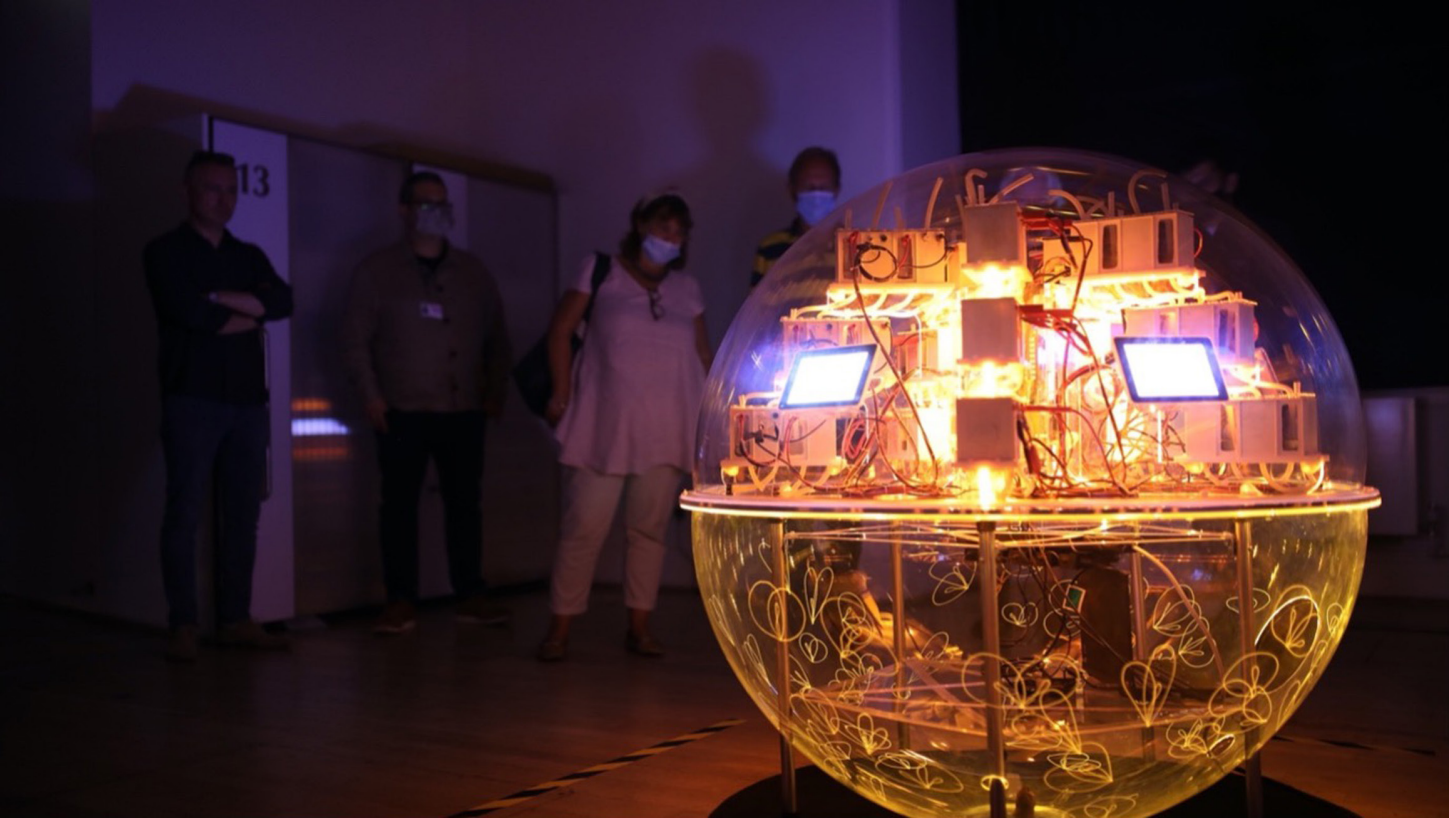
The ALICE installation you see here, is a version of the interface that was exhibited during the Digital Design Week at the Victoria and Albert Museum last year. Courtesy of the ALICE consortium: Ioannis Ieropoulos, Julie Freeman and Rachel Armstrong. ©ALICE, photograph by Julie Freeman.

For household use, bioreactors are hard to read by a layperson and the ‘Active Living Infrastructure: Controlled Environment’, or ALICE *prototype* provides an accessible digital interface (You et al., 2019) that renders the MFC outputs of the system readable and relatable (ALICE, 2019). Through characterful animations called ‘Mobes’ that artistically interpreted biofilm data from the system producing 200 mW/L urine (You et al., 2022), ALICE was also installed as an artwork at the Victoria and Albert Museum (Figure 4) for the Digital Design Weekend in September 2021 and at the Electromagnetic Field festival, Eastnor, in June 2022. Embodying an emerging bio‐digital platform that integrates microbial and artificial intelligences with biological and technical systems, ALICE proposes an architectural synthesis that accessibly indicates to residents how their household waste streams are performing.

#### Scaling MFCs to perform building operations

**FIGURE 5:**
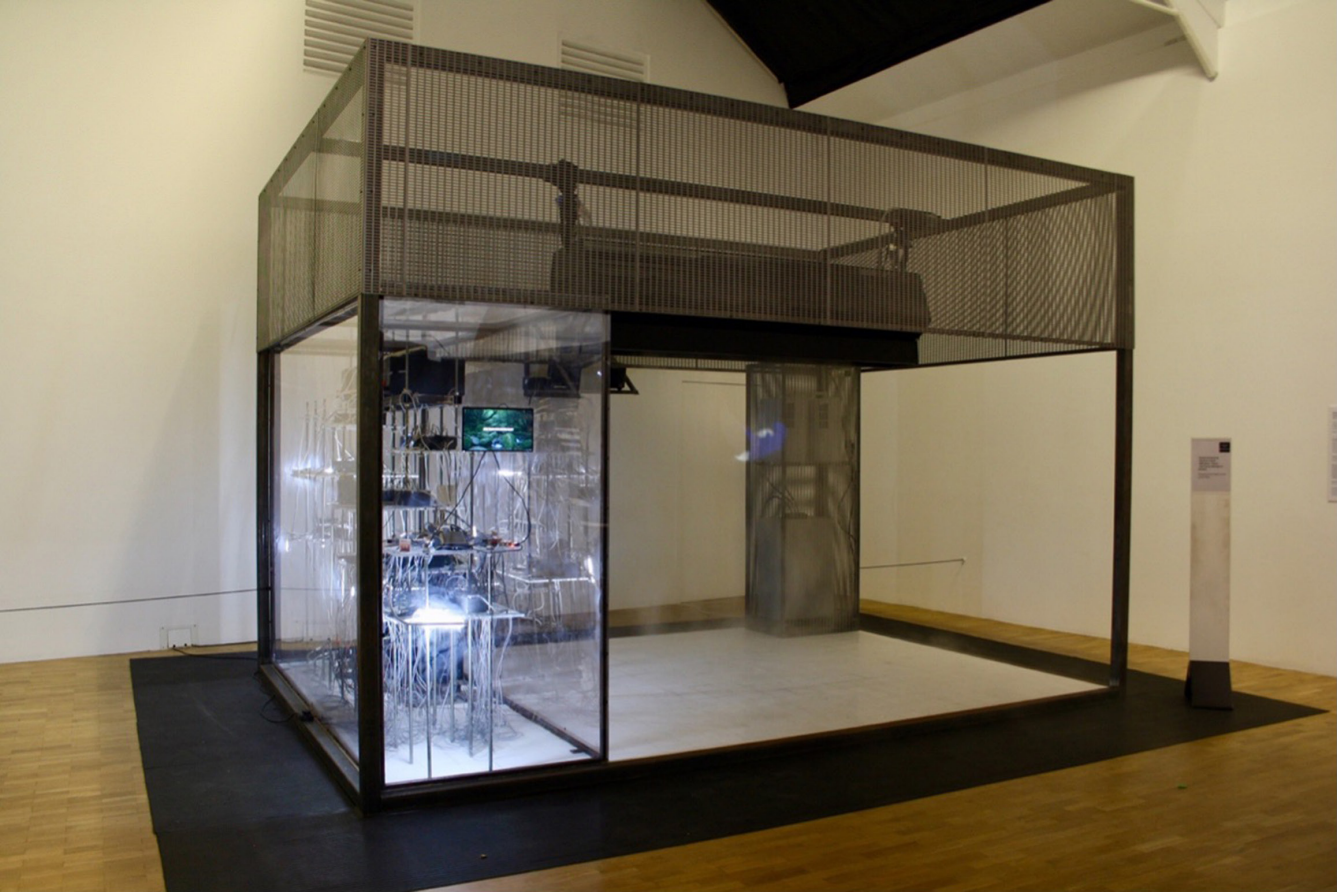
A total of 999 years 13 sqm (the future belongs to ghosts), installation by Rachel Armstrong and Cecile B Evans at the Whitechapel Gallery for the ‘Is This Tomorrow’ group show, Photograph courtesy Rolf Hughes, 2019.

Although the first homes have not yet been built that incorporate MFCs into their building operations, arts and design can again provide a context where different configurations of the arrays can be explored in a public context. The installation *999 years 13 sqm* (*the future belongs to ghosts*), was developed for the *Is this tomorrow?* exhibition at the Whitechapel Gallery, as collaboration between the *Living Architecture* project and artist Cecile B Evans for a group show that was themed on the Whitechapel Gallery's former landmark exhibition *This Is Tomorrow* in 1956 (Bevan, 2019). Providing a dedicated space for housing resident microbes in a stack of MFCs that formed a power‐producing installation, which generated around 200 mW/L urine, the only human presence in the installation could be found in the digital ‘ghosts’ displayed on the screens (Figure 5). Providing a cautionary tale about what might happen if humanity does not establish good relationships with microbes and other life forms, the installation depicted a future time when the only inhabitants of homes and cities are microbes (Timmis & Hallsworth, 2021).

#### Living electronics

Currently, MFCs, PBRs and other bioreactors are made from materials and functional elements that are native to the modern era, that is, they are composed from conventional materials (glass, plastics, steel etc.) and electronic components (silicon, conductive metals, plastics etc.). With advancing scientific developments, electronic components and their casings may be entirely biodegradable and use no Critical Raw Materials. New ways of harvesting and using energy produced by microbes are emerging such as generating power from ambient humidity using protein nanowires (Liu et al., 2020) that—in combination with the convergence of bioelectronics, synthetic biology and electromicrobiology—are enabling the emergence of *living electronics*, where biological agents like biomolecules, cells or cellular communities, are directly incorporated into electronic circuits as electronic components (Dunn, 2020). Microbes use electrical phenomena to enable multicellular communication through using abundant redox‐active molecules and minerals that catalyse global‐scale biogeochemical cycles. Insights into the microbial machinery enabling these redox reactions are being used produce electrical circuits for biotechnological applications with tuneable electrical properties. These living electronic components include wires, capacitors, transistors, diodes, optoelectronic components, spin filters, sensors, logic processors, bioactuators, information storage media and methods for assembling these components into living electronic circuits (Atkinson et al., 2022). To assemble these diverse living electronic elements into living circuits requires ways of spatializing their arrangements on a range of electrode surfaces and non‐conductive surfaces. Adding features and functions to components that are not possible in conventional electronics, these emerging new systems will work synergistically with the environment, using it as a processing adjunct, rather than an extraction site, creating new possibilities for the field of electronics, with some unexpected performance outcomes. Such circuitry will break new ground in the fields of microbiology and electronics, comprising the first fully biodegradable platform for applications with a high commercial potential, and establishing a platform for circular electronics leading to a range of biodegradable electronics components that are powered by organic matter and leave no e‐waste. Importantly, such systems will not operate using the 230 V power supply demanded by industrial development but will create creative limits for resource consumption with respect to electrical performance and creating new potential for bioreactor systems that are not bootstrapped to industrial benchmarks for entry into the marketplace.

#### Services integration

As in the case of biocomposite materials, building services for next‐generation sustainable homes, are likely to link previously separate realms. Specifically, the electricity produced by anaerobic biofilms and PBRs can be combined with microbially powered artificial intelligence (and machine learning) to provide ‘smart’ multi‐utility building systems. Additionally, the direct convergence of the organic and electronics realm, establishes the foundations for a Green Digital Revolution, where using our computers and other ‘smart’ services can help bioremediate our environment (Killeen, 2022) and challenge our assumptions about what kinds of functions a home, or building can provide.

**FIGURE 6:**
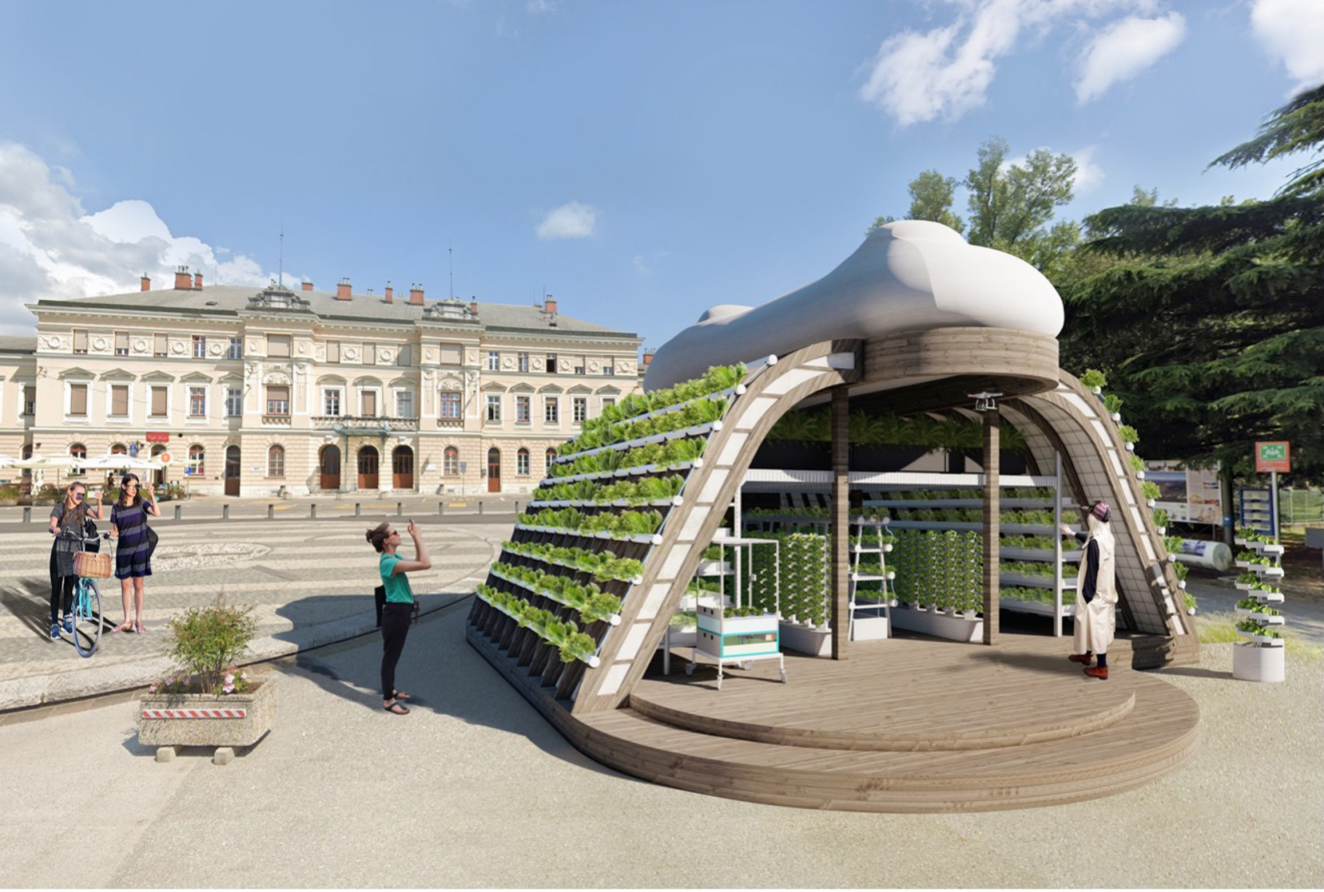
MFC toilet structure made from recycled wood, incorporated into a precision gardening unit with robot‐farmed hydroponically grown plants. Concept for Nova Gorica GO! 2025 festival, rendering by Anna Vershinina, 2023.

For example, a toilet which is presently considered a waste chute that is flushed with fresh water, can be transformed into a site and structure for the production and exchange of a range of microbial ‘goods’ that can be used to make things (substrates for materials, energy, biomolecules and detoxification of waste streams), or even return the microbially processed substances safely to the soil. To unleash the full potential of the microbial technology implicit in these services, new kinds of structures are needed. For example, new structures for housing MFC stacks, can encourage a different attitude towards, and expectations of our toilets (or other wastewater streams) and other sanitary spaces like bathrooms, by incorporating them into gardening functions such as hydroponics systems, where bioavailable nitrogen and phosphate nourishes the root systems of hydroponically grown plants (Figure 6).

For microbially produced materials and building operations to be adopted into mainstream architectural practices, new kinds of services and materials, building typologies and structures must be figured out in detail. While the overall electrical outputs are much lower than compared with fossil fuels, from an ecological perspective, setting these limits on energy consumption at source are creative and invites us to do things that require lots of energy differently. The unlimited energy offered by fossil fuels encourages wasteful patterns of behaviour, which accelerates consumption. Perhaps, the most radical advance suggested by using microbes in our homes is to challenge our basic assumptions about the baselines of what we need to live comfortably and healthily. Imagine the reduced impact of human development if every home that is now connected to a 230 V grid, could operate comfortably on a 12 V battery supply. While this would require innovation in some of the things that we do every day that we solve by consuming a lot of energy, like washing machines and fridges, these same tasks could be done differently, such as using advanced new materials to help with refrigeration and finding alternatives to mechanical agitation like ultrasound to carry out this housework (Armstrong, 2023b).

## TOWARDS ‘LIVING’ CITIES POWERED AND PRODUCED BY MICROBES

Microbially produced materials and building operations have the potential to change how we live and work. Within the innovation process, architectural design plays an important role in enabling cutting edge scientific developments to reach the marketplace through contributions in spatial organization, geometric volumes, interface design, functional programme and social engagement. While microbial technologies and materials are not a panacea for the world's environmental problems, they are advantageous over other kinds of technology for achieving the SDGs since they are directly engaged with biogeochemical cycles and are fundamentally bioremediating. Providing real alternatives to industrial manufacturing processes, a range of SMEs, for example, Biomason, MOGU, are currently developing a range of non‐structural building products that use microbes as a production system. Eventually, new legislation will be needed to incorporate living microbes into the construction process if awareness of the potential for bioremediation within the built environment is raised. With iterations of product lines, costs are competitive as petrochemical products are becoming increasingly unattractive. In modern industries, bio‐based materials are only used when there is a particular technical or cost advantage. Increasing commitments by the commercial sector to sustainability and the SDGs are using biomaterials and other bioproducts to meet their targets. As biological innovation meets downstream demand, a new wave of the Bio Revolution is unfolding across all commercial sectors, and is particularly pertinent for the built environment's huge carbon footprint, with enormous potential impact (Chui et al., 2020). Presently, all biomaterial products are attenuated at point of sale and are validated for applications within established health and safety regulations, with no metabolic impacts beyond their capacity to biodegrade. The biodegradation process alone, however, can make foundational contributions to biodiversity, where decomposition is extremely important to the health of the soils through nutrient cycling that provides a steady, slow‐release source of nitrogen, playing a significant role in carbon storage (Ódor et al., 2006). Furthermore, bioreceptive products that do not incorporate microbes in their production but stimulate their presence, are likely to become a mainstream element for a regenerative architectural toolset. Such developments can be readily adopted by architectural practice through surface patterning on familiar materials (e.g. concrete, wood) that perform conventional architectural functions which also promote secondary colonization by microbes and cryptogamic covers. Globally, cryptogamic covers take up around 3.9 Giga tonnes (Pg) carbon per year, corresponding to around 7% of net primary production by terrestrial vegetation (Elbert et al., 2012). While biocomposite materials are presently not structural, they are compatible with and used in combination with Europe's thriving timber trade that accounts for 9% of Europe's manufacturing Gross Domestic Product (EFI, 2023), where further developments towards the circular bioeconomy will likely enable new kinds of building construction. Additionally, bio‐based material synthesis has become a craft in the design community, which when combined with democratized access to toolsets like 3D printing, laser cutting and CNC milling machines, more site‐specific and vernacular approaches to the construction of space will be possible that are optimized to the resources and needs of each site—thus breaking dependency on global supply chains and activating new forms of social innovation. Companies like BIOHM are even developing new economies of practice using open‐source construction and co‐ownership of buildings, which have the potential for disruptive socio‐economic change (BIOHM, 2023). More ambitiously, commercial technologies like Arborea's BioSolar Leaf will always be much more expensive than seedlings, but on brownfield sites and inhospitable (urban) places that do not readily accommodate the growth of higher plants, technologically supported microalgae are potentially the most viable strategy for tackling air pollution. Since care must be taken with setting up inequalities that arise from the expense of the system, where only wealthy people can afford to have clean air in cities, subsidies will be needed to provide access to clean air for all citizens, so costs could be justified this way.

In making the transition to the next‐generation sustainable practices, the role of the economy itself must also be considered as a potential roadblock to uptake in making the transition to a next‐generation sustainable society. The neoliberal marketplace that presently frames the global trading system has been adopted to varying degrees by member states within the European Union (EU) (Germany, and the Netherlands), which advocates for free‐market capitalism, deregulation, privatization and reduced government intervention—where competition in the marketplace decides on the success of any breakthrough. Other European countries like Sweden, Denmark and Finland adopt more mixed economic systems that combine elements of neoliberalism with government intervention in the economy, providing subsidies and other kinds of support for cultural adoption of preferred ways of living and working. In the neoliberal framework, significant returns anticipated by investors and the disruptive tend to be provided by technologies that support the established marketplace, for example, centralized energy systems. Since microbial technologies are disruptive, then alternative forms of commerce and leadership at the level of the EU will be needed to attain proposed sustainable development by targeted grants, and early piloting of new approaches, which potentially activate other forms of economy activated such as the domestic/commons economic axis that empowers communities over businesses.

Of particular interest, is considering the *microbial commons* itself as a form of economy. Experienced as a ‘living’ combined utilities infrastructure within our homes, liquid household waste, like urine and grey water, can be transformed into valuable resources (electricity, biomass, water, reclaiming phosphate from washing‐up liquids and removing poisonous gases from the air)—a biological Midas touch! Based on electrons as the unit of exchange, multiple tasks within the apparatus can be performed through metabolic transformations (*microbial housework*), which are compatible with the overall carrying capacity of a household system and combined with renewables to charge 12 V battery systems or participate in the smart grid trade (Yaagoubi & Mouftah, 2015). With the prospect of waste being re‐used in the household, utilities bills can be reduced along with the amount of untreated waste we release into the environment. Establishing an economics‐first principle, the transactional metabolic system at the basis of the microbial commons creates new kinds of value which is shared by all who carry out the work‐of‐life. Importantly, the different microbial units that comprise domestic systems will enable those that are not typically regarded as economically productive in a capitalist economy (e.g. the elderly) to participate economically within the microbial commons. Re‐centring the site of value creation within the domestic sphere, our homes become wealth‐generators, where inhabitants have choices to make about how they use this ecological resource. Perhaps, they will reduce their own living costs but maybe too, they can donate some of their well‐earned resource (formerly called ‘waste’) to help others.

Although there is still some way to go before microbial technological solutions (materials and building operations) can be fully integrated into a single house or building—but that time is not far away if reduced environmental impacts are our strategic priority. The hardest part to altering our impacts is changing our thinking, our habits and our ideas about what living a ‘good life’ means. Whether we like it, or not, the rules for living on this planet have changed and better understanding what microbes can do, is key to establishing an era of next‐generation sustainable development, where the human species can become a fundamentally bioremediating and enlivening force within the biosphere.

## RELEVANCE FOR SUSTAINABLE DEVELOPMENT GOALS AND GRAND CHALLENGES

The incorporation of microbes into building operations, bioreceptive surfaces and microbially made materials—whether locally applied within the household, or scaled to community and city dimensions—relates to several SDGs (*microbial aspects in italics*), outlined below:

- *Goal 3. Ensure healthy lives and promote well‐being for all at all ages* (*improve health, reduce preventable disease and premature deaths*). Incorporating bioreceptive surfaces, microbially made materials and bioreactors, can actively cultivate beneficial microbes into our homes and cities, are likely to have overall beneficial effects on human health, specifically via the probiotic balance between the *human microbiome*, the *microbiome of the built environment* and the *urban microbiome* (Brownell, 2020; King, 2014). By re‐enriching these ecological communities, life‐promoting interactions and environmental services can be fostered that promote the health of soils, increase biodiversity beyond the conventional focus on plants and animals to improve the overall health of cities and their human inhabitants. All these beneficial environmental effects promote well‐being and good health with economic consequences for health budgets.
- *Goal 6. Ensure availability and sustainable management of water and sanitation for all* (*assure safe drinking water, improve water quality, reduce pollution, protect water‐related ecosystems, improve water and sanitation management*). Microbial fuel cells (MFCs) play a crucial role in simultaneously biodegrading organic matter in wastewater, removing pathogen loads and bioremediating toxins (e.g. heavy metals), while *converting the chemical energy within the bonds into electrical energy to provide low‐power renewable bioelectricity*, which reduces the overall cost of wastewater treatment through safe and local effluent disposal.
- *Goal 7. Ensure access to affordable, reliable, sustainable and modern energy for all* (*ensure access to clean, renewable, and sustainable energy, and increase energy use efficiency*). MFCs not only generate low‐power renewable energy, which is more than a natural way of providing electrical energy, but also establishes an off‐grid emergency communications platform through powering 6GWi‐Fi networks in the immediate term. As the MFC platform is further refined, new units for construction will become increasingly available being stackable, lightweight and versatile in their design enabling new kinds of structures and, ultimately novel building typologies. Currently, a 1000 unit MFC stack comprises 46 modules of 5 L geometrical volume each, which is 230 L total volume. With a 50 L header tank and peripherals, this volume becomes 300 L. With smaller MFC modules, two tiers of 22 MFCs/module can be produced reducing the total number of modules to 23, which would be 115 L total volume (~190 L with tanks and peripherals). The power density of MFC modules is also set to reach 1 mW/mL feedstock. Scaled to 1000‐MFC units at 1–2 mW/MFC, 1‐2 W and 24–48 Wh can be reasonably expected for 1 day, with 168 Wh‐3 1 year 36 Wh per week, 720 Wh – 1.4 kWh for 1 month and 8.7–17.5 kWh for ar, taking a significant step towards resource circularity for human settlements raising the possibility of 12 V domestic lifestyles that go beyond survival and bioremediate our surroundings, while meeting—and perhaps 1 day surpassing—the basic expectations of a modern existence (Gajda et al., 2018). In the medium to longer term, a ‘living’ utilities system based on smart toilet networks that generate bioelectricity are possible, which, when coupled to low power electronics enable a Green (bioremediating) Digital Revolution.
- *Goal 8. Promote sustained, inclusive and sustainable economic growth, full and productive employment, and decent work for all* (*promote economic growth, productivity and innovation, enterprise and employment creation*). Since the work of life generates feedstock for microbes (urine, black water, grey water and liquid organic wastes), this forms a domestic currency of electrons. By exchanging our ‘wastes’ for electrons, cleaned water, biomolecules and de‐toxification processes within the home, even those who are not economically productive within a capitalism value system can make valuable contributions within a domestic economy.
- *Goal 9. Industry and innovation* (*promote economic growth, productivity and innovation, enterprise and employment creation*). The development of new microbially based construction materials and building operations processes is pure innovation in industry and will lead to the creation of new enterprises likely to become a major driver of economic growth. Crucially, it will be economic growth in a key area of sustainability and in response strategies to the global warming crisis: *necessity is the mother of invention*!
- *Goal 11. Sustainable cities and communities* (*build resilient infrastructure, promote inclusive and sustainable industrialization and foster innovation*). MFCs in particular form the basis for ‘smart’ sewerage and plumbing systems with biosensing potential, that is, they can detect the composition of their liquid contents, and provide electrical signals that linearly correspond with targeted contaminants. From a microbially produced materials perspective, the ability to use local organic wastes as cost‐effective fabrics reduces reliance on fossil fuel derivatives while increasing the value of waste streams that are available to all (owing to the base value of all raw materials). In combination, microbial solutions establish the potential of paradigm shifting building technologies (services and materials) that are cultured and grown, rather than mined, burned or extracted, fundamentally changing the impact of the built environment on our living world to reach new levels of sustainability and resilience against climate change.
- *Goal 12. Responsible consumption and production* (*ensure sustainable consumption and production patterns*). By valorizing waste streams, microbial technologies (materials and systems) enable an implementable system for a circular economy founded on zero waste, renewable energy and where ‘customers’ become ‘users’, which does not exclusively rely on, or is defined by, human actions alone.
- *Goal 13. Take urgent action to combat climate change and its impacts* (*reduce greenhouse gas emissions, mitigate consequences of global warming, develop early warning systems for global warming consequences, improve education about greenhouse gas production and global warming*). The incorporation of microbial technologies (materials and systems) into our homes, communities and cities can improve resilience, preparedness and responsiveness of the built environment for climate adaptation through resource circularity, bioremediating waste streams, setting limits to energy consumption and inviting innovation for low‐powered solutions for carrying out the activities of daily life. Forming the operational basis for community transactions through access to the microbial commons, such microbial technologies establish a platform for a circular resource economy enabling new kinds of urban exchanges.
- *Goal 15. Life on Land* (*protect, restore and promote sustainable use of terrestrial ecosystems, sustainably manage forests, combat desertification and halt and reverse land degradation and halt biodiversity loss*). Outputs from microbial technologies (materials and services) can be integrated with established building systems in retrofit, new build or cultural heritage buildings and connected to existing utilities such as rainwater collection systems, and regenerative energy sources via the smart energy grid (solar, wind). The resultant versatile ‘Living’ buildings systems infrastructure is bioremediating and can be deployed across a range of vital services from hydroponics to sanitation, green energy and environmental sensing. In this way, microbial technologies can be implemented to form a ‘Living’ utilities system optimizing energy consumption, while having regenerative impacts on soil health, land fertility and biodiversity, thereby, rendering urban spaces more bio‐positive—that is, life‐promoting.
- *Goal 17. Partnerships for the goals* (*strengthen the means for implementation and revitalize the Global Partnership for Sustainable Development*). Offering a range of new materials, building services and modes of producing work, microbial technologies establish the principles for breakthrough innovation at all stages within the built environment with benefits to producers, and consumers through a bioremediated, low energy environment that ultimately replenishes land fertility, increasing biodiversity. Heralding an era of change the potential of paradigm shifting building technologies based on microbial technologies that are cultured and grown in our homes and communities alongside us, fundamentally change the impact of the built environment on our living world to reach new levels of sustainability and resilience against climate change through new sources of bioenergy, sanitation, circularity for natural resources, increased soil and water health. Neither optional extras nor an architectural fashion, installing microbial technologies in our homes, buildings, and cities will, literally, save lives and invite partnerships from all levels of the building industry, utilities and land management services. At a time of climate emergency, escalating fuel prices and the displacement of peoples from war, having access to basic utilities as a combined processing system can provide clean water, shelter, power and sanitation, which maintains a basic liveability made possible through the microbial commons to generate freely available materials, even in extremis.

## Endnote

^1^
The ethical grey zone here refers both to the pathogenic potential of commensals like *E. Coli* but also to their lack of backbone which is a criterion for their legal protection under Directive 2010/63/EU, and revised Directive 86/609/EEC on the protection of animals used for scientific purposes.
